# Co-production of fully renewable medium chain α-olefins and bio-oil *via* hydrothermal liquefaction of biomass containing polyhydroxyalkanoic acid

**DOI:** 10.1039/c8ra07359g

**Published:** 2018-10-08

**Authors:** Tao Dong, Wei Xiong, Jianping Yu, Philip T. Pienkos

**Affiliations:** National Bioenergy Center, National Renewable Energy Laboratory 15013 Denver West Parkway Golden CO 80401 USA tao.dong@nrel.gov

## Abstract

Medium chain-length linear α-olefins (*mcl*-LAO) are versatile precursors to commodity products such as synthetic lubricants and biodegradable detergents, and have been traditionally produced from ethylene oligomerization and Fischer–Tropsch synthesis. Medium chain-length polyhydroxyalkanoic acid (*mcl*-PHA) can be produced by some microorganisms as an energy storage. In this study, *Pseudomonas putida* biomass that contained *mcl*-PHA was used in HTL at 300 °C for 30 min, and up to 65 mol% of *mcl*-PHA was converted into *mcl*-LAO. The yield and quality of the bio-oil co-produced in the HTL was remarkably improved with the biomass rich in *mcl*-PHA. Experiments with extracted *mcl*-PHA revealed the degradation mechanism of *mcl*-PHA in HTL. Overall, this work demonstrates a novel process to co-produce *mcl*-LAO and bio-oil from renewable biomass.

## Abstract 2

Co-production of fully renewable medium chain α-olefins and bio-oil by hydrothermal liquefaction.

## Graphical abstract

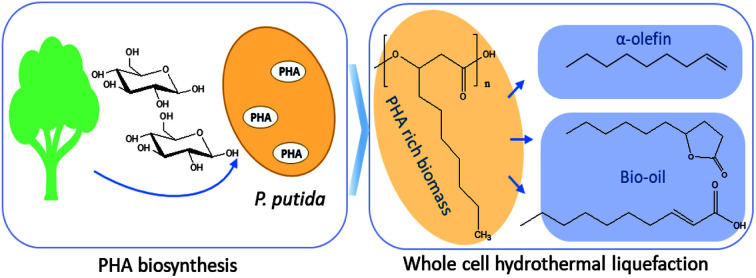

## Introduction

Due to their terminal functionality, linear α-olefins (LAO) are extremely versatile and valuable precursors to produce many commodity chemicals. The short chain (C_2–4_) LAO can be used as feedstock to produce polyethylene, polypropylene and acrylonitrile. Medium chain-length LAO (*mcl*-LAO) are of particular interest because they can be used as “drop-in” fuels that are compatible with the existing engine systems and transportation infrastructure.^1^*mcl*-LAO are also widely used as co-monomers (C_5_–C_8_) in polymer production, and to produce poly α-olefins (PAO) as base stocks for synthetic lubricants for automotive and industrial applications. Long chain-length LAO can be converted into valuable fatty alcohols to produce biodegradable detergents,^2^ and environmentally friendly drilling fluid base stocks. The global LAO market size was valued at USD 8.26 billion in 2016 and is expected to grow at a compound annual growth rate (CAGR) of 4.8% for the next 10 years.^3^

Petroleum derived feedstocks have been traditionally used for LAO production, mainly *via* Fischer–Tropsch synthesis or catalytic oligomerization of ethylene to produce a mixture of largely linear products of C_4_–C_20_+ with even carbon number chain length. The processes require non-renewable resource and are energy intensive. With increasing energy demand and growing concern about carbon emissions, there has been increasing global interest in producing fuels and chemicals through sustainable approaches, such as from renewable biomass.

Hydrothermal liquefaction (HTL) is a thermal process operating under subcritical water environment that converts wet biomass into four phases: an oil phase (bio-oil), a solid phase (biochar), a gas phase (largely CO_2_), and an aqueous phase (containing substantial amounts of water soluble organics and inorganics). Water is usually the only solvent in this process. HTL bypasses the energy intensive step of drying biomass, simplifies downstream processing and reduces cost. Therefore, HTL has been widely applied in microbial biomass conversion and upgrading.^4^ Recently, chemical assisted liquefaction has also been employed to produce value-added bio-derived products at high yields.^5,6^ Here we developed a novel approach to produce renewable *mcl*-LAO based on the early studies that propylene can be produced from polyhydroxybutyric acid (PHB) as a thermal degradation product, where the hydroxybutyrate units undergo an intramolecular β-elimination to produce crotonic acid, which is then converted into propylene *via* decarboxylation.^7^ A recent research also reported propylene production from cyanobacterial biomass rich in PHB *via* HTL,^8^ consistent with these thermochemical reactions. We hypothesized that *mcl*-LAO could be produced from medium chain-length polyhydroxyalkanoic acid (*mcl*-PHA) *via* a similar series of reactions. The work described here demonstrates that renewable *mcl*-LAO can be co-produced with bio-oil in a non-catalytic HTL process from the bacterium *Pseudomonas putida* biomass rich in *mcl*-PHA.

## Materials and methods

### Chemicals

1-Undecene and 1-tridecene were purchased from Alfa Aesar (MA, USA). PHB, benzoic acid, 1-heptene, 1-nonene, benzoic acid and 3-hydroxybutyric acid were purchased from Sigma-Aldrich (MO, USA). Methyl ester of 3-hydroxyoctanoic acid (3-OH C_8_), 3-hydroxydecanoic acid (3-OH C_10_), 3-hydroxydodecanoic acid (3-OH C_12_) and 3-hydoxytetradecanoic acid (3-OH C_14_) were purchased from Matreya LLC (PA, USA). All solvents and reagents were either of HPLC grade or analytical reagent grade.

### Cultivation of *Pseudomonas putida*

*P. putida* KT2440 was grown as published by Sun *et al.*^9^ The inoculum was grown in a 500 mL shake flask with 100 mL growth medium supplemented with 10 g L^−1^ glucose, pH 6.8 and incubated in a shaking incubator at 31 °C with 200 rpm. The shake flask medium contained per liter: 4.7 g (NH_4_)_2_SO_4_, 0.8 g MgSO_4_·7H_2_O, 12 g Na_2_HPO_4_·7H_2_O, 2.7 g KH_2_PO_4_, 10 g glucose and 1 g nutrient broth (DIFCO, USA, 231000). After 12 hours growth the inoculum culture was transferred into a BioFlow 3000 fermenter with 4 L growth media containing 10 g L^−1^ glucose with initial OD (600 nm) of 0.15. The fermentation medium was similar to shake flask medium except the Na_2_HPO_4_ concentration was increased to 18 g L^−1^, the KH_2_PO_4_ concentration was increased to 4.05 g L^−1^, and the medium was supplemented with 8 mL of trace minerals solution as described by Sun *et al.*^9^ The pH was controlled at 6.8 using NH_4_OH (14%) for first 11 hours to build biomass then it was controlled by KOH (4N) afterwards to induce PHA production. Oxygen was kept at ≥40% saturation using 0.5–1 L min^−1^ air flow and by variable agitation. At 10 h 3.5 mL of antifoam (Sigma SE 204, 15%) was added to the fermenter to prevent foaming. After 10.5 h fermentation 1.8 L of broth was withdrawn to harvest the first batch of biomass. This volume was replaced with 2 L of fermentation medium without (NH_4_)_2_SO_4_ to continue the fermentation. From 11 h glucose feed (600 g L^−1^ glucose and 10 g L^−1^ MgSO_4_·7H_2_O) was started at the rate of 5 mL h^−1^. The feeding rate was increased to 25 mL h^−1^ at 24 h. The second batch of biomass was harvested at 30 h by withdrawing 2 L of broth, and that volume was replaced with 1 L of fermentation medium without (NH_4_)_2_SO_4_. At 47 h 150 mL of glucose feed was added. At 49 h all the biomass was harvested by centrifuging at 10 000 rpm (Sorvall Lynx 6000). Biomass samples for HTL studies were frozen and stored at −20 °C. Biomass samples for PHA extraction were lyophilized before solvent extraction.

### Extraction of *mcl*-PHA

PHA was extracted from freeze-dried *P. putida* biomass powder using dichloromethane (DCM) three times (biomass to DCM = 1 : 15, w/v) for 4 hours at room temperature.^10^ The solution was filtered through a glass fiber filter to remove cell debris. The *mcl*-PHA resin was obtained by removing solvent using rotary evaporation. Then the resin was dissolved in DCM (resin to DCM = 1 : 10, w/v). The solution was added dropwise into a cold methanol solution (DCM/methanol = 1 : 10, v/v)^10,11^ and kept at 4 °C for 2 hours. The solvent was decanted to obtain precipitated resin. The residual solvent was removed by evaporation in a vacuum oven at 40 °C overnight.

### HTL process

HTL reactors were made with 316 stainless steel with 4 in. length of 1/2 in. O.D. tube with a wall thickness of 0.065 in. A cap is placed on one end, and the other end is fitted with an 18 in. length of 1/8 in. O.D. tube, with a wall thickness of 0.028 in., connected to a high-pressure valve. In a typical experiment 5 mL of biomass slurry (20 wt% DCW) was loaded into a reactor.^12^ The slurry loading was selected such that 95% of the reactor volume will be occupied by liquid at reaction conditions. The air in the headspace of the reactor was replaced with helium by repeated cycles of evacuation and charging with helium. A 140 psi of helium was remained serving as an internal standard for the quantification of gas yields.

HTL reactions were carried out by placing the reactors vertically in a fluidized sand bath, and the temperature was maintained at 300 °C for 30 min. After the reaction, the reactors were removed from the sand bath and immersed in a cold-water bath for about 30 min to quench the reaction. The reactors were placed in ambient temperature for up to 3 hours to allow the liquid and gas phase to equilibrate. The gas phase was collected into air bags for analysis. The gas bags were directly hooked up to and analyzed by an Agilent 490 micro-GC with Molecular Sieve 5A, PoraPLOT Q, CP-Sil 5CB, and CP-Wax 52CB columns for He, N_2_, H_2_, CO, CO_2_, and C_1_–C_4_ hydrocarbons.^13^

### HTL product recovery

**Fig. 1:**
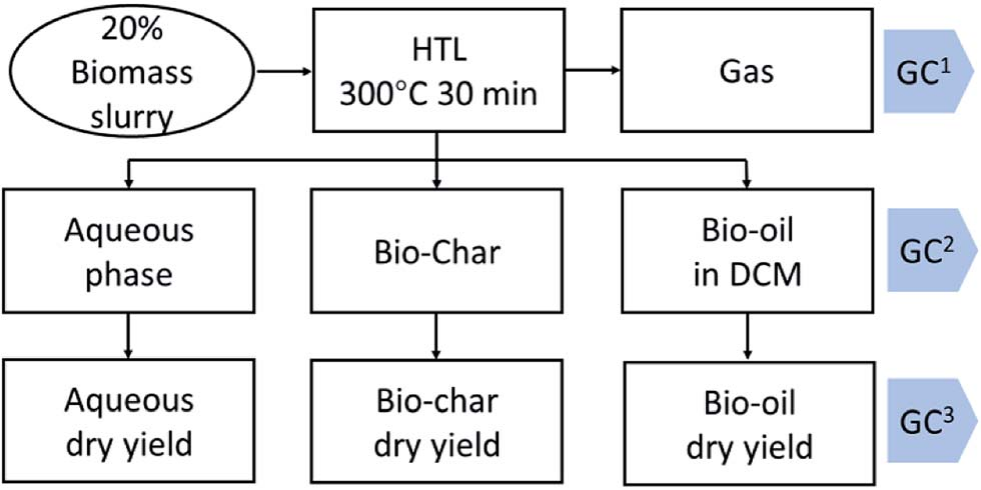
Small scale HTL experiment setting up (the gas phase was analyzed by GC^1^; the bio-oil in dichloromethane (DCM) was analyzed by GC^2^; after DCM evaporation the bio-oil was analyzed by GC^3^).

The mixture in the reactor was transferred to a separatory funnel and the reactor was rinsed with DCM and DI water to ensure complete transfer. The funnel was shaken vigorously to extract bio-oil into the DCM phase. Then the phases were allowed to separate under gravity. The DCM and aqueous phase were sequentially filtered to remove biochar. The obtained DCM phase was transferred into a 10 mL volumetric flask to make up to 10 mL using DCM. One microliter of DCM phase was injected into a GC for analysis. Then an aliquot of DCM phase was transferred into a pre-weighted tube. The DCM was evaporated under a nitrogen flow for 2 hours. Then the obtained bio-oil was evaporated in a vacuum oven under 40 °C for 2 hours to get a gravimetric yield. An aliquot of aqueous phase was freeze-dried to get the dry weight for aqueous phase yield. The general HTL product recovery scheme is illustrated in Fig. 1.

### Compositional analysis of biomass

The composition of biomass was analyzed using protocols described previously.^14^ Ash content was quantified by burning weighted samples in a muffle furnace.^15^ Carbohydrate content was quantified by hydrolyzing the biomass with sulfuric acid and analyzing on a Thermo Scientific Dionex ICS 5000 system equipped with pulsed amperometric detection (PAD). Fatty acids were converted into fatty acid methyl esters (FAMEs) and analyzed by gas chromatography-flame ionization detection (GC-FID) on an Agilent 7890. Carbon, hydrogen and nitrogen are determined by an external laboratory (Hazen Labs, Golden, CO) on a Flash EA 1112 Series Elemental Analyzer. The technique is the classical Dumas method, with thermal conductivity detection. The method is described in ASTM D5373 (coal) and ASTM D5291 (petroleum products). Briefly, weighed samples are combusted in oxygen at 1000 °C. The combustion products are swept by a helium carrier gas through combustion catalysts, scrubbers and a reduced copper tube. All gases are then separated on a chromatography column and measured by thermal conductivity detection (TCD) detection. Oxygen was assumed to account for the bulk of the remaining sample mass balance. Higher heating value (HHV; MJ kg^−1^) was estimated using Dulong's formula.^16^

1 HHV = 0.3383C + 1.422(H–O/8)

### PHA analysis

PHA content was analyzed by a modified methylation derivation method.^17^ Briefly, 10 mg sample, 1 mg of benzoic acid internal standard and 1 mL of derivatization agent BF_3_/MeOH were sequentially fed into a silanized glass vial. The vial was sealed and heated at 80 °C for 20 hours. The contents in the vial was transferred into a 10 mL volumetric flask and made up to 10 mL with DCM. Then the solution was transferred into a 20 mL glass vial and mixed with 3 mL of water to wash out acid. The DCM phase was transferred to another vial with anhydrous Na_2_SO_4_ and Na_2_CO_3_. One microliter of solution was injected into GC for analysis.^17^

### GC analysis

One microliter of sample in DCM was injected into a gas chromatography-flame ionization detection/mass spectra (GC-FID/MS) on an Agilent 7890 A GC equipped with a polyarc (Activated Research Company, MN, USA) FID detector. The inlet temperature was 260 °C. Oven temperature started at 50 °C, hold for 2 min, ramped at 10 °C min^−1^ to 120 °C, hold for 1 min, ramped at 10 °C min^−1^ to 220 °C, hold for 1 min, ramped at 25 °C min^−1^ to 300 °C, and hold for 2 min. The injected compounds were separated by an Agilent HP-5MS column (30 m × 0.25 mm i.d. × 0.25 μm film thickness) and detected by the FID and MS, respectively. Peaks were identified by NIST11 library. Quantification of the compounds was based on a 5-point calibration curve obtained from FID.

## Results and discussion

### Production of propylene from PHB *via* HTL

It has been reported that PHB can be converted into propylene under HTL condition.^8^ We conducted experiments with pure PHB at 300 °C for 30 min in our HTL setting and confirmed the propylene product in the gas phase after the experiment. Up to 43 mol% of hydroxybutyrate units was converted into propylene. Recently, bio-derived propane has garnered increased attention for its potential to reduce greenhouse gas footprint.^18^ Propane can be produced from propylene *via* catalytic hydrogenation. We tested for *in situ* production of propane by conducting HTL of PHB in the presence of hydrogen. In this experiment we purged 750 psi of hydrogen instead of He into the reactor with PHB and performed HTL at 300 °C for 30 min. Propane was indeed detected in the gas phase after the reaction, indicating the feasibility to produce a fully renewable propane by this approach. However, the propane yield was low (1.2 mol%), presumably because there was no catalyst for the hydrogenation and the condition was not optimal for hydrogenation. We think that a higher propane yield can be achieved by applying a hydrogenation catalyst either in the HTL process, or in a sequential downstream upgrading step. Since propylene is a gas under HTL process, the catalyst can be placed in the head space in the reactor to avoid being fouled by other compounds (liquid or solid phase) in the HTL. Alternatively, the propylene can also be easily separated from liquid phase after HTL to be upgraded through a downstream catalytic bed.^19,20^ Further studies will be needed to test these ideas.

Based on the high propylene yield from PHB in HTL process we hypothesized that *mcl*-LAO can be produced from *mcl*-PHA *via* the similar pathway. *mcl*-PHA is naturally produced by some microbes and could be a renewable feedstock. Since there is no commercially available *mcl*-PHA, we decided to produce *mcl*-PHA using *P. putida* fermentation.

### Production of *mcl*-PHA using *P. putida*

**Fig. 2:**
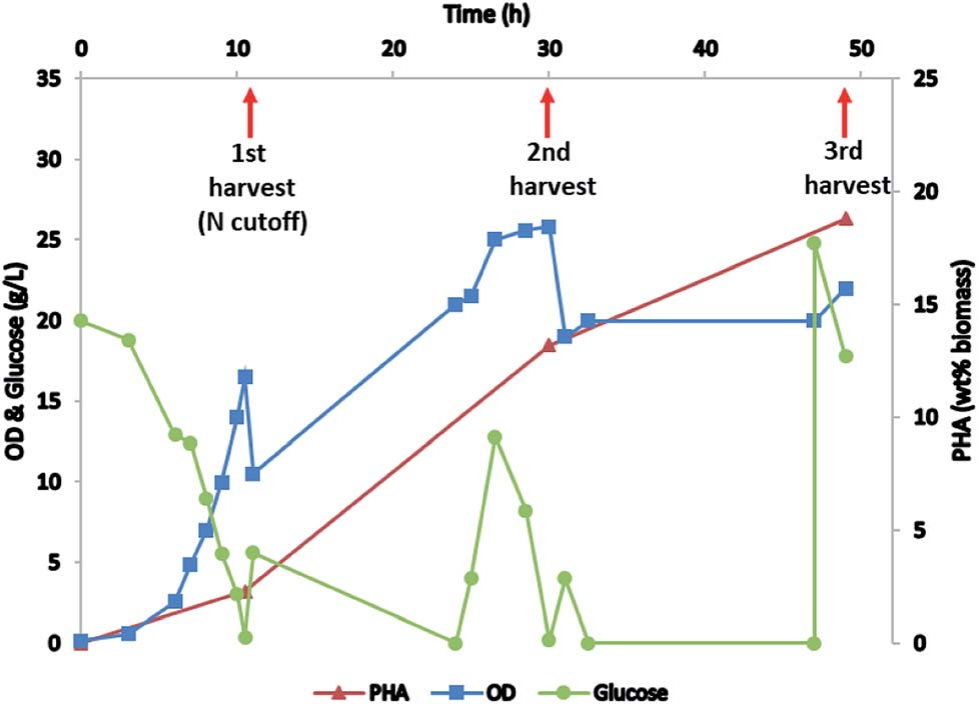
Cultivation of *P. putida* to accumulate *mcl*-PHA.

*P. putida* is known for its capacity to accumulate high amounts of *mcl*-PHA when grown on glucose^9^ or other carbon sources including lignin^17^ under nitrogen deplete conditions. Here we fed the *P. putida* with glucose for biomass production and *mcl*-PHA accumulation. Biomass was built up in the initial phase of growth by maintaining a high ammonia concentration through the use of NH_4_OH to control pH. In the second phase of the fermentation, nitrogen was depleted from the medium by replacing NH_4_OH with KOH for pH control (Fig. 2). Biomass was harvested at three different growth stages (early stage with nitrogen replete condition, middle stage at the beginning of nitrogen depletion, and late stage with nitrogen depleted) and *mcl*-PHA content was analyzed, confirming that the *mcl*-PHA content increased by nitrogen depletion strategy (Fig. 2).

### HTL of biomass to co-produce *mcl*-LAO and bio-oil.

The total PHA in the early stage was 2.3% DCW, and it increased to 18.6% at the end of the fermentation, with 3-hydroxydecanoic acid as the dominant monomer (Table 1). During the fermentation, carbon and hydrogen content increased, while nitrogen content decreased, due to the nitrogen depletion. The total higher heating value (HHV) of the biomass increased from 17.0 to 20.9 indicating an increase in energy content of the biomass with the accumulation of PHA. The total carbohydrate and fatty acid content declined slightly with nitrogen depletion.

**Table 1:** Summarizes the composition of the biomass^a^

Harvested biomass	Total PHA %	3-OH C_8_%	3-OH C_10_%	3-OH C_12_%	FAME %	Carbohydrates %	Ash %	Nitrogen %	Carbon %	Hydrogen %	Oxygen %	HHV MJ kg^−1^
1^st^ harvest	2.3	0.0	1.5	0.8	4.1	12.7	14.0	11.1	43.0	6.6	39.3	17.0
2^nd^ harvest	13.3	3.2	8.9	1.2	2.8	13.4	10.8	7.0	45.2	6.9	40.9	17.8
3^rd^ harvest	18.6	4.9	12.2	1.5	2.1	10.9	9.1	5.7	49.8	7.4	37.0	20.9

a 3-OH C_8_: 3-hydroxyoctanoic acid; 3-OH C_10_: 3-hydroxydecanoic acid; 3-OH C_12_: 3-hydroxydodecanoic acid.

**Fig. 3:**
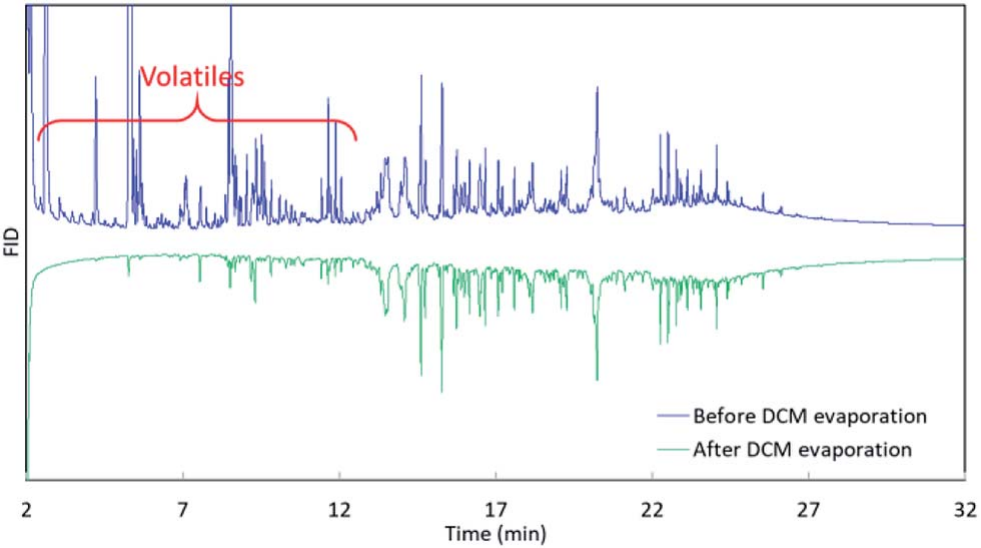
GC analysis of collected bio-oil before and after solvent evaporation.

The 3^rd^ harvest biomass listed in Table 1 was used for HTL to evaluate if *mcl*-LAO and bio-oil could be co-produced. We followed the classic small scale HTL sample preparation process, in which bio-oil was recovered by DCM rinsing/extraction and solvent evaporation (Fig. 1). An aliquot of bio-oil sample was dissolved in DCM and analyzed by GC (Fig. 1, GC^3^), but no *mcl*-LAO was found (Fig. 3, green chromatograph). We speculated that the volatile *mcl*-LAO products might be lost during DCM evaporation, which is a method that has been routinely applied in the classic small scale HTL sample preparation.^4,21^ Then, we analyzed the DCM solution before the evaporation (Fig. 1. GC^2^) and found considerable amounts of volatile compounds (Fig. 3, blue chromatography), confirming our concern that volatile compounds could be lost during solvent evaporation. Thus, solvent evaporation in a small scale HTL study can lead to underestimation of bio-oil yield and failure to identify potentially valuable volatile compounds. In principle, solvent extraction is not necessary at pilot scale or larger, because the produced bio-oil can be separated spontaneously from aqueous phase under gravimetric settling.^22^ We believe this is a potential issue that has been overlooked for traditional small scale HTL experiments, and that published bio-oil yields may be artifactually low and opportunities for exploitation of volatile products may have been missed by others.

In this case, we identified and quantified volatile olefin products, such as 1-heptene, 1-nonene and 1-undecene, by analyzing DCM solution before evaporation. These *mcl*-LAO with odd carbon chain numbers (*n* − 1) are believed to arise from the C_*n*_ 3-hydroxyalkanoic acids units *via* decarboxylation (Table 2). In contrast to the traditional olefin process, such as ethylene oligomerization, in which only even number LAOs can be produced, LAOs with odd carbon numbers can be produced by PHA degradation. These odd carbon LAOs will not necessarily serve as direct replacements for petrochemical LAOs but their properties may allow the development of novel applications.

**Table 2:** *mcl*-LAO yields after HTL of *P. putida* biomass and extracted PHA^a^

3-Hydroxyalkanoic acid	Produced *mcl*-LAO	*mcl*-LAO yield (% of biomass/material)		
		1^st^ harvest	3^rd^ harvest	Extracted PHA
3-Hydroxyoctanoic (C_8_)	1-Heptene (C_7_)	0.0	1.5	4.7
3-Hydroxydecanoic (C_10_)	1-Nonene (C_9_)	0.4	6.1	24.6
3-Hydroxydodecanoic (C_12_)	1-Undecene (C_11_)	0.2	0.8	2.4

a Alkene yields are based on the AFDW of starting materials.

Then, we used the 1^st^ harvest biomass (with minimum *mcl*-PHA) and extracted PHA for HTL under the same conditions and the LAO yields were summarized in Table 2. For the 1^st^ harvest biomass the *mcl*-LAO yield was very low, corresponding to the low *mcl*-PHA content (2.3%) in that batch of biomass. The *mcl*-LAO yield from extracted PHA was the highest among the three experiments. These results support our hypothesis that *mcl*-PHA can be the precursor for *mcl*-LAO production.

Besides the production of *mcl*-LAO, bio-oil, aqueous phase, bio-char and gas were produced *via* the HTL process. The yields of these fractions were summarized in Table 3. The bio-oil yield was significantly higher for the 3^rd^ harvest biomass that was rich in *mcl*-PHA, while the yields of non-volatile aqueous phase and char were significantly lower for this biomass, indicating a higher conversion efficiency from biomass to bio-oil. The carbon and nitrogen analysis on the aqueous phase showed that the aqueous phase contained about 50% of the total nitrogen from the starting biomass for both materials. The aqueous phase generated from the 1^st^ harvest biomass and 3^rd^ harvest biomass contained 24% and 15% of the total carbon from the starting materials, respectively. During HTL carbohydrate will generate hydroxymethylfurfural, acetic acid, *etc.*, while protein will be degraded into amino acid, peptides and other derivatives.^23^ Most of these water-soluble degradation products will end in the aqueous phase.^4^ Since the carbohydrate and FAME contents for 1^st^ harvest and 3^rd^ harvest were similar (Table 1), the remarkable increase of bio-oil yield in 3^rd^ biomass is very likely due to the presence of *mcl*-PHA. In the gas phase, the dominant compound was carbon dioxide, with trace amount of hydrogen and methane. The 3^rd^ harvest biomass generated much more carbon dioxide, as expected from decarboxylation of alkenoic acids.

**Table 3:** HTL results for *P. putida* biomass^a^

	Bio-oil yield%	Aqueous yield%	Char yield%	Gas yield%	*mcl*-LAO yield%
1^st^ harvest	23.4 ± 2.1	45.6 ± 4.0	10.7 ± 1.5	4.8 ± 3.5	0.8 ± 0.1
3^rd^ harvest	32.6 ± 1.3	28.1 ± 3.5	7.4 ± 1.0	10.2 ± 1.9	8.3 ± 0.2

a Bio-oil, aqueous phase (non-volatile), gas and alkene yields are based on AFDW.

The carbon, hydrogen and nitrogen contents in the obtained bio-oil samples are shown in Table 4. Bio-oil derived from the 3^rd^ harvest biomass contained higher levels of carbon and hydrogen resulting in a higher heating value (HHV), while the nitrogen content was reduced by 31% compared to the 1^st^ harvest. Thus the quantity and quality of bio-oil were both improved by increasing *mcl*-PHA content in the biomass. HTL has been recognized as a composition-independent process to convert biomass into bio-oil. However, recent studies have demonstrated that the composition of biomass can significantly affect the bio-oil yield and quality.^21^ Increasing bio-oil yield and quality by tuning up the composition of biomass might be a promising approach to further drive down biofuel cost.

**Table 4:** Carbon, hydrogen and nitrogen analysis on bio-oil products

Bio-oil samples	Nitrogen %	Carbon %	Hydrogen %	Oxygen %	HHV MJ kg^−1^
1^st^ harvest (N replete)	8.6	71.9	9.3	10.2	35.7
3^rd^ harvest (N deplete)	5.9	73.6	10.0	10.5	37.3

PHA is a promising biopolymer,^24^ and a feedstock for biofuel production.^17^ Previously, both processes involve biomass dehydration and solvent extraction of the PHA from the biomass. The extracted PHA in the latter process requires thermal-degradation to produce unsaturated fatty acids (such as decenoic acid), which can be converted into hydrocarbon biofuels *via* catalytic upgrading.^17^ However, dehydration of microbial biomass for extraction is energy-intensive,^25^ extraction requires solvent recycling and can complicate downstream processing, and noble metal catalysts may not be economical for biofuel production. In this experiment, we applied HTL as an alternative approach to integrate downstream processes, demonstrating co-production of bio-oil and *mcl*-LAO in one step. The produced *mcl*-LAO can be easily separated from the bio-oil, due to the high volatility of these compounds. Moreover, the yield and quality of bio-oil was remarkably improved with the presence of *mcl*-PHA in the starting biomass. As such, the production of value-added *mcl*-LAO in addition to high-quality bio-oil *via* a simple and integrated pathway could help drive the economics.

### GC-MS analysis of bio-oil derived from *mcl*-PHA rich biomass

Bio-oil produced from HTL process usually consists of many compounds. The result of GC-MS analysis on the bio-oil obtained from 3^rd^ harvest biomass is summarized in Table 5. Alkenes with odd carbon number, such as heptene, nonene, and undecene are the major compounds that can be detected by GC-MS. 3-Decenoic acid, 2-decenoic acid and γ-decalactone could be derived from 3-hydroxydecanoic acid unit in the *mcl*-PHA, but we were concerned about the complexity of interactions among various compounds in the starting biomass therefore decided to extract PHA from the biomass to get a clearer picture on the fate of the *mcl*-PHA in the HTL.

**Table 5:** GC-MS analysis of bio-oil derived from *mcl*-PHA rich biomass before solvent evaporation^a^

No.	RT (min)	Compound	M^+^(*m*/*z*)	Formula	Relative concentration (area %)
1	2.61	Heptene	98	C_7_H_14_	13.4
2	3.05	1,3-Diazine	80	C_4_H_4_N_2_	1.1
3	4.21	Methylpyrazine	94	C_5_H_6_N_2_	2.1
4	5.35	Nonene	126	C_9_H_18_	49.1
5	5.60	2,6-Dimethyl pyrazine	108	C_6_H_8_N_2_	2.4
6	7.09	2-Ethyl-5-methyl pyrazine	122	C_7_H_10_N_2_	0.7
7	7.57	*N*-Isobutylacetamide	115	C_6_H_13_NO	0.5
8	8.45	1,4-Undecadiene	152	C_11_H_20_	1.3
9	8.54	Undecene	156	C_11_H_20_	6.7
10	9.03	(*Z*)-Cycloundecene	152	C_11_H_20_	0.8
11	9.35	1-Hexylcyclopentene	152	C_11_H_20_	0.9
12	9.52	2,9-Undecadiene	152	C_11_H_20_	0.6
13	9.83	1-Acetylpyrrolidine	113	C_6_H_11_NO	0.6
14	11.64	Cycloundecene	152	C_11_H_20_	1.0
15	11.87	1-Tridecene	182	C_13_H_26_	0.9
16	13.59	3-Decenoic acid	170	C_10_H_18_O_2_	3.2
17	14.20	*trans*-2-Decenoic acid	170	C_10_H_18_O_2_	3.1
18	14.62	γ-Decalactone	170	C_10_H_18_O_2_	2.1
19	15.26	4-Hexylphenol	178	C_12_H_18_O	0.8
20	15.30	*N*-(2-phenylethyl)acetamide	163	C_10_H_13_	1.0
21	16.67	Azacyclotridecan-2-one	197	C_12_H_23_NO	0.7
22	20.26	Hexadecanoic acid	256	C_16_H_32_O_2_	5.6
23	22.03	Oleic acid	282	C_18_H_34_O_2_	0.8
24	22.28	Hexadecanamide	255	C_16_H_33_NO	0.7

a Only identified compounds are shown in this table. Relative concentration was determined by area of each compounds using a polyarc FID.

### Thermal degradation of *mcl*-PHA in HTL

Extracted *mcl*-PHA resin, water, and deuterated toluene (internal standard) were fed into tube reactors for HTL at 300 °C in a sand bath. The reactors were quenched with ice water at 5, 10, 15 or 30 min, respectively. Then the contents were extracted by DCM into a volumetric flask for GC analysis. Since the 3-hydroxydecanoic acid was the major compound (66%) in the extracted *mcl*-PHA resin, we tracked the fate of this compound and its derivatives during the process to illustrate the thermal degradation mechanism of mcl-PHA in HTL.

**Fig. 4:**
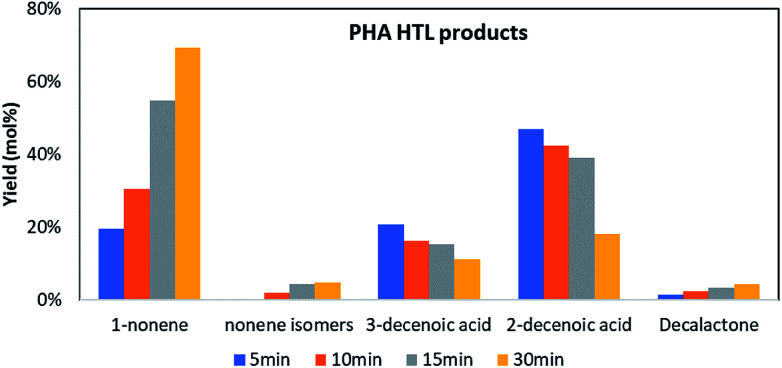
PHA thermal degradation products in HTL process.

In this HTL process 5 major derivatives that are related to 3-hydroxydecanoic acid were identified and quantified, and they are 1-nonene, nonene isomers (2-nonene, 3-nonene, *etc.*), 3-decenoic acid, 2-decenoic acid and decalactone. As shown in Fig. 4, the concentration of 2-decenoic acid and 3-decenoic acid decreased, while the yield of 1-nonene, nonene isomers and decalactone increased during the HTL process. A final yield for 1-nonene of 69 mol% was observed at 300 °C after 30 min. The final yield for 3-decenoic acid and 2-decenoic acid was 11 mol% and 18 mol%.

**Fig. 5:**
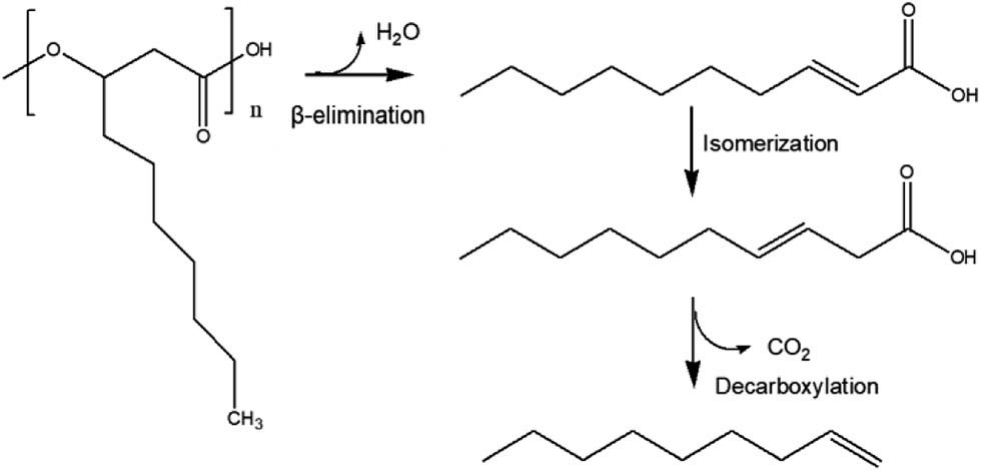
Proposed *mcl*-PHA degradation pathway.

Based on a previous report, in which PHB thermal degradation was studied by experimental and density functional theory (DFT) analysis,^26^ we propose here a similar *mcl*-PHA thermal degradation pathway (Fig. 5): During the HTL process, the depolymerization of *mcl*-PHA took place initially by an intramolecular β-elimination to produce 2-decenoic acid, which was then converted into 3-decenoic acid *via* isomerization, followed by decarboxylation to produce 1-nonene as the final product. Other reaction pathways (such as hydrolysis of *mcl*-PHA to form 3-hydroxyfatty acid, followed by dehydration of 3-hydroxyfatty acid to produce unsaturated fatty acids) might also take place simultaneously.

In this study we have demonstrated that the *mcl*-PHA can be converted directly into *mcl*-LAO *via* HTL, and the residue alkenoic acids can be collected into the bio-oil phase after the HTL. The residue alkenoic acid can be converted into drop-in hydrocarbon biofuel *via* catalytic upgrading. This study provides a simplified and integrated process that has potential to reduce the cost of biofuel production.

Unlike the HTL bio-oil from other feedstocks, the bio-oil produced from *mcl*-PHA biomass had a pleasant smell. We believe that the fragrance can be attributed to γ-decalactone and γ-octalactone that were identified in the bio-oil. γ-decalactone is an aroma compound present naturally in many fruits and fermented products. It is particularly important in the formulation of peach, apricot, and strawberry flavors.^27^ Many synthetic γ-lactones have been utilized as artificial flavors. γ-lactones are also versatile platform molecule that can be used for the production of liquid fuels and chemicals.^28,29^ γ-lactones can serve as precursors for biopolymers *via* ring-opening polymerization,^30^ offering a novel route for renewable biopolymers.^31^ γ-lactone products have been produced from microbial processes,^32,33^ but the low productivity remains a hurdle to overcome. This research provides an alternative approach to produce γ-lactone from biomass *via* a scalable thermal chemical pathway.

Besides these major compounds, other derivatives such as dienes, alcohols and aldehydes were also detected after the HTL of mcl-PHA (data not shown), indicating other unknown reaction mechanisms. The thermal degradation of *mcl*-PHA in HTL process comprises of a series of complex pathways, which might be able to be controlled by catalysts and reaction kinetics to produce preferred chemicals.

## Conclusions

In this study we have demonstrated co-production of *mcl*-LAO and bio-oil from biomass *via* non-catalytic HTL. Up to 65 mol% of *mcl*-PHA was converted into *mcl*-LAO. Co-products and reaction intermediates such as unsaturated fatty acids, olefin isomers and lactones, were also identified. This process represents a simpler and more energy efficient route to produce *mcl*-LAO, and possibly also long chain LAO from corresponding PHA in biomass, and could contribute to lower cost biofuels.

## Conflicts of interest

There are no conflicts to declare.

## Supplementary Material

## References

[cit1] Lennen R. M., Pfleger B. F. (2013). Curr. Opin. Biotechnol..

[cit2] Dry M. E. (2002). Catal. Today.

[cit3] Grand View Research, Alpha Olefin Market Analysis By Product, (1-Butene, 1-Hexene, 1-Octene, 1-Decene, 1-Dodecene), By Application (Polyethylene, Detergent Alcohol, Synthetic Lubricants), By Region, And Segment Forecasts, 2018 – 2025, https://www.grandviewresearch.com/industry-analysis/alpha-olefins-market

[cit4] Miao C., Chakraborty M., Dong T., Yu X., Chi Z., Chen S. (2014). Bioresour. Technol..

[cit5] Pierson Y., Chen X., Bobbink F. D., Zhang J., Yan N. (2014). ACS Sustainable Chem. Eng..

[cit6] Zhang J., Yan N. (2016). Green Chem..

[cit7] Clark J. M., Nimlos M. R., Robichaud D. J. (2015). J. Phys. Chem. A.

[cit8] Wagner J., Bransgrove R., Beacham T. A., Allen M. J., Meixner K., Drosg B., Ting V. P., Chuck C. J. (2016). Bioresour. Technol..

[cit9] Sun Z., Ramsay J. A., Guay M., Ramsay B. A. (2006). Appl. Microbiol. Biotechnol..

[cit10] Wampfler B., Ramsauer T., Rezzonico S., Hischier R., Köhling R., Thöny-Meyer L., Zinn M. (2010). Biomacromolecules.

[cit11] Huijberts G. N., Eggink G., de Waard P., Huisman G. W., Witholt B. (1992). Appl. Environ. Microbiol..

[cit12] Duan P., Savage P. E. (2011). Ind. Eng. Chem. Res..

[cit13] Iisa K., French R. J., Orton K. A., Dutta A., Schaidle J. A. (2017). Fuel.

[cit14] Dong T., Knoshaug E. P., Davis R., Laurens L. M. L., Van Wychen S., Pienkos P. T., Nagle N. (2015). Algal Res..

[cit15] Van Wychen S. and Laurens L. M. L., Determination of Total Solids and Ash in Algal Biomas – Laboratory Analytical Procedure (LAP), Golden, CO, 2013

[cit16] Biller P., Ross a. B. (2011). Bioresour. Technol..

[cit17] Linger J. G., Vardon D. R., Guarnieri M. T., Karp E. M., Hunsinger G. B., Franden M. A., Johnson C. W., Chupka G., Strathmann T. J., Pienkos P. T., Beckham G. T. (2014). Proc. Natl. Acad. Sci. U. S. A..

[cit18] Johnson E. (2017). Biofuels, Bioprod. Biorefin..

[cit19] Argo A. M., Odzak J. F., Lai F. S., Gates B. C. (2002). Nature.

[cit20] Camacho-bunquin J., Aich P., Ferrandon M., Bean A., Das U., Dogan F., Curtiss L. A., Miller J. T., Marshall C. L., Hock A. S., Stair P. C. (2017). J. Catal..

[cit21] Leow S., Witter J. R., Vardon D. R., Sharma B. K., Guest J. S., Strathmann T. J. (2015). Green Chem..

[cit22] Elliott D. C., Hart T. R., Schmidt A. J., Neuenschwander G. G., Rotness L. J., Olarte M. V., Zacher A. H., Albrecht K. O., Hallen R. T., Holladay J. E. (2013). Algal Res..

[cit23] Teymouri A., Adams K. J., Dong T., Kumar S. (2018). Fuel.

[cit24] Choi J., Lee S. Y. (1999). Appl. Microbiol. Biotechnol..

[cit25] Dong T., Knoshaug E. P., Pienkos P. T., Laurens L. M. L. (2016). Appl. Energy.

[cit26] Clark J. M., Pilath H. M., Mittal A., Michener W. E., Robichaud D. J., Johnson D. K. (2016). J. Phys. Chem. A.

[cit27] An J., Joo Y., Oh D. (2013). Appl. Environ. Microbiol..

[cit28] Tang X., Zeng X., Li Z., Hu L., Sun Y., Liu S. (2014). Renewable Sustainable Energy Rev..

[cit29] Wang D., Hakim S. H., Alonso D. M., Dumesic J. A. (2013). Chem. Commun..

[cit30] Chalid M., Heeres H. J., Broekhuis A. A. (2011). J. Appl. Polym. Sci..

[cit31] Hong M., Chen E. Y. (2016). Nat. Chem..

[cit32] Feron G., Dufosse L., Pierard E. V. A., Bonnarme P., Quere J. L. E., Spinnler H., De Recherches L. (1996). Appl. Environ. Microbiol..

[cit33] Mota M., Teixeira J. A. (2005). Biotechnol. Lett..

